# Evidence of Age-Related Hemodynamic and Functional Connectivity Impairment: A Resting State fMRI Study

**DOI:** 10.3389/fneur.2021.633500

**Published:** 2021-03-23

**Authors:** Eleftherios Kavroulakis, Nicholas J. Simos, Thomas G. Maris, Ioannis Zaganas, Simeon Panagiotakis, Efrosini Papadaki

**Affiliations:** ^1^Department of Radiology, School of Medicine, University of Crete, University Hospital of Heraklion, Heraklion, Greece; ^2^Department of Electrical and Computer Engineering, Technical University of Crete, Chania, Greece; ^3^Computational Bio-Medicine Laboratory, Institute of Computer Science, Foundation for Research and Technology – Hellas, Heraklion, Greece; ^4^Department of Medical Physics, School of Medicine, University of Crete, University Hospital of Heraklion, Heraklion, Greece; ^5^Department of Neurology, School of Medicine, University of Crete, University Hospital of Heraklion, Heraklion, Greece; ^6^Department of Internal Medicine, University Hospital of Heraklion, Heraklion, Greece

**Keywords:** aging, resting state functional MRI, intrinsic connectivity contrast, time shift analysis, cerebral blood flow

## Abstract

**Purpose:** To assess age-related changes in intrinsic functional brain connectivity and hemodynamics during adulthood in the context of the retrogenesis hypothesis, which states that the rate of age-related changes is higher in late-myelinating (prefrontal, lateral-posterior temporal) cerebrocortical areas as compared to early myelinating (parietal, occipital) regions. In addition, to examine the dependence of age-related changes upon concurrent subclinical depression symptoms which are common even in healthy aging.

**Methods:** Sixty-four healthy adults (28 men) aged 23–79 years (mean 45.0, *SD* = 18.8 years) were examined. Resting-state functional MRI (rs-fMRI) time series were used to compute voxel-wise intrinsic connectivity contrast (ICC) maps reflecting the strength of functional connectivity between each voxel and the rest of the brain. We further used Time Shift Analysis (TSA) to estimate voxel-wise hemodynamic lead or lag for each of 22 ROIs from the automated anatomical atlas (AAL).

**Results:** Adjusted for depression symptoms, gender and education level, reduced ICC with age was found primarily in frontal, temporal regions, and putamen, whereas the opposite trend was noted in inferior occipital cortices (*p* < 0.002). With the same covariates, increased hemodynamic lead with advancing age was found in superior frontal cortex and thalamus, with the opposite trend in inferior occipital cortex (*p* < 0.002). There was also evidence of reduced coupling between voxel-wise intrinsic connectivity and hemodynamics in the inferior parietal cortex.

**Conclusion:** Age-related intrinsic connectivity reductions and hemodynamic changes were demonstrated in several regions—most of them part of DMN and salience networks—while impaired neurovascular coupling was, also, found in parietal regions. Age-related reductions in intrinsic connectivity were greater in anterior as compared to posterior cortices, in line with implications derived from the retrogenesis hypothesis. These effects were affected by self-reported depression symptoms, which also increased with age.

## Introduction

The majority of imaging studies on the effect of healthy aging on cerebral hemodynamics have examined cross-sectional changes in brain metabolism using SPECT (1, 2) or FDG-PET (3–11) and cortical perfusion using Arterial Spin Labeling (ASL) (12–21). With certain exceptions (6, 7), the majority of studies reported decreased cerebral metabolism with age which tends to be more pronounced in the frontal lobe, especially in rostral and ventral prefrontal regions, the temporal pole, and caudate (8–11).

Studies with ASL found a global gray mater CBF decline of about 7.7% per decade of age (18) or annual decline by 0.4–0.7% (12, 17, 19, 20). Age-related regional CBF reductions were, also, found by ASL, predominantly in frontal lobes (12, 13, 19). Other ASL studies demonstrated opposite CBF changes in different brain regions. For instance, Preibisch et al. (21) reported lower CBF among 25 adults >50 years of age as compared to a group of 19 younger adults (<45 years) in the superior parietal and occipital cortices and higher CBF in superior and middle temporal cortex. More recently, Zhang et al. (17) found evidence for both increasing (in the putamen, orbitofrontal cortex, cingulate gyrus, insula, and basal temporal cortex) and decreasing regional cerebral perfusion with age (in dorsal and medial prefrontal regions, inferior and superior parietal lobule, temporal pole, and cerebellum). The areas noted for age-related reductions in CBF were virtually identical to those found by Chen et al. (15).

Studies on the effect of healthy aging on the integrity of cerebral white matter have focused on major WM tracts through Diffusion Tensor Imaging (DTI) and tractography techniques (22–31). This topic relates to a long-standing proposal—known as the retrogenesis hypothesis—according to which fibers which complete their myelination late during brain development are more susceptible to age-related changes or pathology than earlier-myelinated fibers (32–34). Primary sensory and motor regions in the occipital and (anterior) parietal lobes are amongst the earliest to reach microstructural maturity, whereas structural maturation presumably continues into adulthood in prefrontal and lateral-posterior temporal regions, hosting association cortices (35, 36). In a previous study using T2 relaxometry, we have reported greater reductions in myelin water content measured within normal appearing white matter (NAWM) in prefrontal as compared to occipital brain regions (37). Hemisphere differences in white matter characteristics may have confounded earlier reports on age-related effects. Such hemisphere differences have not been explored in sufficient anatomical detail, with the majority of existing reports focusing on white matter characteristics in regions implicated in language functions and reporting the expected L>R asymmetry in fractional anisotropy (FA) (38) and Magnetic Transfer Ratio (MTR) (39).

Moreover, microstructural characteristics and function of white matter fiber tracts are expected to be both physiologically (i.e., directly through common neurovascular processes) and functionally (i.e., indirectly) related to the activation of nearby cortical areas (40). It follows that age-related changes in the integrity and perfusion of the cerebral white matter may parallel changes in ongoing cortical function. Resting-state functional MRI (rs-fMRI) is a non-invasive imaging technique, using blood oxygenation level-dependent (BOLD) signal, that has been widely used to investigate changes in functional brain organization in normal aging. In the majority of studies brain function has been explored through estimates of functional connectivity within predefined brain networks. The default mode network (DMN) is perhaps the most consistently found to display reduced within-network connectivity with aging (41–46). Regions most commonly associated with the DMN include medial prefrontal, posterior cingulate cortex (PCC) extending into the precuneus, the inferior parietal lobule, and the middle temporal gyrus extending into the temporal pole. The ventral attention (or salience) network has also been reported to show reduced within-network connectivity with age by some studies (47). This network comprises the insula, anterior cingulate (ACC), and inferior orbital cortex.

In addition, it has been reported that reduced connectivity within major networks with age may be paralleled by increased connectivity between networks (48). This trend for reduced modularity across the life span highlights the need to explore connectivity changes independent of predefined connectivity networks. There is very limited work on age-related changes using a whole-brain voxel approach which is in principle more sensitive to individual differences in global connectivity patterns. One such method relies on the Intrinsic Connectivity Contrast (ICC) which does not require setting a priori seed regions in order to estimate connectivity strength. Only recently Galiano et al. (49) reported reduced ICC in prefrontal regions, insula, and temporal pole in older adults paralleled by ICC increases in inferior occipitotemporal and occipitoparietal regions. Age-related reductions in ICC have been also reported by Bagarinao et al. (50) in prefrontal, insular, inferior parietal, and basal temporal regions and, also, in the putamen and cingulate gyrus.

Resting-state functional MRI could provide evidence not only about neural activity, but also about cerebral hemodynamic status, by using time-shift analysis, a promising new method that has been used to assess hemodynamics in previous studies (51–59). According to this method, the hemodynamic transfer speed (hemodynamic lag or lead times) is evaluated by using the temporal shift of low-frequency BOLD signal fluctuations of rs-fMRI, and correlates with regional brain perfusion (54, 60). This approach can also be used to assess coupling between hemodynamic status and functional connectivity at the same time, by identifying voxels that display relatively high levels of functional connectivity with other brain areas coupled with hemodynamic lead (and conversely voxels that display relatively low levels of functional connectivity with other brain areas coupled with hemodynamic lag).

Further, the degree of functional connectivity of a given cortical region with other brain areas, as measured by ICC, is directly linked to intrinsic neurophysiological activity in this region (61). In turn, increased regional neurophysiological activity would place higher metabolic demands and increased regional blood flow to meet these demands (62). Whether these hypothesized compensatory changes in perfusion are sufficient to maintain baseline neurophysiological function in the affected brain regions, and preserve intrinsic connectivity remains unexplored. To our knowledge only one study has systematically assessed age-related changes in perfusion-connectivity coupling reporting decreasing trends with age in superior temporal, orbitofrontal, and cingulate cortex, using fMRI data based on ASL perfusion (49).

To address these issues, we examined changes in hemodynamic response and associated intrinsic connectivity associated with healthy aging, by assessing voxel-level hemodynamic and functional connectivity indices derived from a single technique (rs-fMRI), using time-shift analysis. Specifically, we explored potential implications of the retrogenesis hypothesis, as well as hemisphere effects, in age-related changes in functional brain indices, including hemodynamic-functional connectivity coupling. For this, we integrated voxel-based estimates of regional hemodynamic transfer speed (hemodynamic lag or lead times) associated with regional brain perfusion, with indices of functional connectivity within prefrontal (ventromedial and dorsolateral prefrontal cortex), temporoparietal, and occipital cortices. Additional cerebrocortical regions were also examined following earlier studies that focused either on network connectivity (precuneus, temporal pole, cingulate gyrus, insula), thalamus and basal ganglia. Given the prevalence of late-onset depression symptoms in the aging population (63) and previous reports of significant associations between subclinical depression symptoms and functional connectivity (64–66) we included self-reported depression symptomatology as a covariate in these analyses.

## Materials and Methods

### Participants

The sample consisted of 64 adults (28 men) averaging 45.02 years of age (*SD* = 18.81, range 23–79 years) who had completed an average of 13.37 years of formal education (*SD* = 4.74, range 5–20 years). Persons 60 years or older were recruited as part of a research project on aging and age-related cognitive decline conducted in the District of Heraklion, Crete, Greece. The participant pool consisted of 259 self-referred elders recruited through ads in local media to be tested for “memory and other cognitive difficulties.” Older participants were deemed free from neurological or psychiatric disorder (including Mild Cognitive Impairment of any type according to the International Working Group on Mild Cognitive Impairment criteria (IWG) (67) based on comprehensive neuropsychiatric and neuropsychological evaluation. Younger persons were recruited through local ads, were screened negative for history of neurological or psychiatric disease and did not report persistent cognitive difficulties in daily life. In addition, all participants were found to be free of any structural abnormalities on MRI other than a mild load (<10) of small (<1 cm) white matter hyperintensities (WMH), that are quite common in older persons. For group-level analyses participants were assigned to two age groups (20–44, ≥45 years) consisting of 22 and 42 participants, respectively.

Self-reported symptoms of depression were recorded using the Greek version of the Hospital Anxiety and Depression Scale (HADS) (68) with a widely employed cutoff for clinically significant anxiety or depressive symptomatology of 7/8 points. Written informed consent as approved by the Clinical Research Ethics Board of the University Hospital of Heraklion was obtained from all participants.

As expected, participants in the younger group had completed more years of formal education (17.75, *SD* = 1.06 vs. 12.12, *SD* = 4.64, *p* < 0.001), and reported less severe depression symptoms (HADS Depression subscale: 3.58, *SD* = 1.83 vs. 6.61, *SD* = 2.16, *p* < 0.001).

### MR Image Acquisition

All MRI exams were performed on a 1.5 T MR scanner (Vision/Sonata hybrid System, Siemens, Erlangen). Conventional MR imaging protocolcomprised three sequences: (1) a 3D T1-weighted (T1w) magnetization prepared rapid gradient echo (MPRAGE) (TR/TE/TI: 1,570/2.73/800 ms, 1.5 × 1.5 mm in-plane resolution, 1 mm slice thickness/0 mm gap, 160 oblique axial space filling slices), (2) a 2D T2w TSE (TR/TE: 5,000/98 ms, 4 mm slice thickness/1.6 mm gap, 21 oblique axial slices), and (3) a 2D T2w turbo fluid attenuated inversion recovery (FLAIR; TR/TE/TI: 9,000/120/2,600 ms, 4 mm slice thickness/1.6 mm gap, 21 oblique axial slices). Axial sections were acquired parallel to the plane connecting the anterior and posterior commissures (AC-PC line).

Resting-state functional MRI was derived from a T2^*^-weighted, fat-saturated 2D-FID-EPI sequence with repetition time (TR) 2,300 ms, echo time (TE) 50 ms, field of view (FOV) 192 × 192 × 108 (x, y, z). Acquisition voxel size was 3 × 3 × 3 mm, whole brain scans consisted of 36 transverse slices acquired parallel to the plane passing through the AC-PC line with 3.0-mm slice thickness and no interslice gap.

### fMRI Data Preprocessing and Denoising

Each BOLD time series consisted of 150 dynamic volumes (the first three were ignored in all subsequent analyses). The fMRI images were co-registered, smoothed, and normalized and to the MNI space using SPM12. In terms of signal denoising, Gray Matter, White Matter, and cerebrospinal fluid signal (CSF) mean signals were regressed out of all voxel time series in order to mitigate their effects on BOLD time courses. These preprocessing steps were applied to the data used for voxel level ICC. For Time Shift Analysis (TSA), only the CSF signal was regressed out of the BOLD fMRI time courses, while all the remaining steps were applied as described above.

### Time Shift Analysis

Time Shift Analysis maps were calculated, as described in several other studies (52, 53, 55–60) using in house MATLAB scripts. Firstly, a mask of the major venous sinuses was created based on the standard brain. The reference BOLD time series was calculated as the mean of all voxel time series included in the venous mask. Then, voxel-wise cross-correlations were calculated in reference to this regressor for lags of −3 TRs to 3 TRs (or −6.96 to 6.96 s). The percentage of voxels with positive (representing hemodynamic lag ≥1 TR) or negative time delay values (representing hemodynamic lead ≥1 TR) across all 90 regions of the automated anatomical atlas (AAL) atlas was calculated for each subject. This entails the computation of the lagged versions of each voxel time series (−3 TR to +3 TR) and of the correlation coefficient of each lagged version of the time series with the reference signal. The lag-value corresponding to the highest correlation coefficient is then assigned to each voxel as its time shift/delay value. A broader range of signal delay (e.g., −6 TRs to +6 TRs) was not selected mainly because of the relatively high TR-value attainable in the present study. Inspection of TSA histograms within each ROI revealed moderately skewed distributions in most cases. Thus, instead of averaging across all voxels we computed binary TSA masks for either positive or negative voxel time delay values [for a similar approach see Luppi et al. (69)]. The main dependent variable used in statistical analyses was percentage of voxels within a given ROI that displayed TSA-values >2.3 s (1 TR lead) or smaller than −2.3 s (1 TR delay). The global average positive or negative TSA-value, respectively, was included in the ANCOVA models as an additional covariate to account for individual differences in overall hemodynamic response.

### Voxel-Wise Functional Connectivity

Voxel-wise global connectivity was assessed through the ICC, an estimate of the degree of association between the time-series of a given voxel with all the remaining voxels in all 90 regions of the AAL atlas. Intrinsic connectivity contrast is based on the graph-theoretical measure of degree. Degree signifies the number of other nodes connected to each node, while the calculation of ICC on a weighted graph, takes into account the connectivity strengths of all connections present for each node. Specifically, a voxel's ICC-value is computed as the mean of that voxel's time series correlation values with all other voxels' time series, squared. The explicit calculation of full voxel-to-voxel functional connectivity matrices in fMRI datasets such as the present is computationally prohibitive, mostly due to software RAM usage limitations. MATLAB code for the calculation of ICC maps using Singular Value Decomposition, similar to the implementation found in CONN (70) and is freely available online. Intrinsic connectivity contrast maps for all subjects were obtained for all voxels within the AAL regions using a global mask, ICC-values were square rooted so as to retain the same scale as common functional connectivity values. In a manner similar to the derivation of TSA-related dependent variables we computed separate binary masks for low and high ICC-values on a subject level. Voxels below the 25th or above the 75th percentile of a subject's voxel ICC-values were assigned to the low and high ICC maps, respectively, and the percentage of low and high voxel ICC-values within each region was computed for each subject.

### Voxel-Wise Coupling Between Hemodynamics and Intrinsic Connectivity

We also computed two separate overlay masks for each ROI representing coupling between hemodynamics and intrinsic connectivity: one mask comprised voxels displaying relatively low ICC coupled with TSA-values >1 TR (hemodynamic lag), whereas the second mask comprised voxels displaying relatively high ICC coupled with TSA-values <1 TR (hemodynamic lead). Spatial voxel-wise clustering as well as spatial smoothing with a three-dimensional Gaussian filter of 3 × 3 × 3 voxels were applied to the final overlap mask. Spatial clustering entailed the discarding of all voxels that were not surrounded by at least ten neighboring voxels with a high value (>75th percentile). In this manner, most scattered and singular voxels were discarded from the final image. The smoothed images were produced for display purposes and were not used in the results discussed below. The resulting dependent variables were: (a) percentage of voxels within each ROI displaying relatively high ICC-values coupled with hemodynamic lead and (b) percentage of voxels within each ROI displaying relatively low ICC-values coupled with hemodynamic lag.

### ROI Selection

Time Shift Analysis, intrinsic connectivity contrast, and ICC–TSA coupling indices were computed for the 10 main anatomical regions covering prefrontal, temporoparietal, and occipital cortices used to examine anterior-posterior gradients in age-related trends. Additional regions were also examined as motivated by previous aging research, namely primary sensory (auditory, somatic) and motor cortices, supplementary motor cortex, paralimbic cortex (anterior and posterior subdivisions of the cingulate gyrus and insula), temporal pole, basal ganglia, and thalamus. As listed in Table 1 measurements were obtained for a total of 22 anatomical regions separately in each hemisphere.

**Table 1:** List of selected anatomical regions, corresponding ROIs according to the AAL atlas, and brain partitions used to assess anterior-posterior age-related gradients.

**Anatomical region**	**Abbreviation**	**ROI label in AAL atlas**
**Prefrontal**		
Inferior frontal gyrus	IFG	11,12,13,14,15,16
(pars orbitalis, pars opercularis, pars triangularis)		
Ventromedial prefrontal cortex	vmPFC	5,6,25,26
(superior frontal gyrus: orbital and medial)		
Dorsolateral prefrontal cortex	dlPFC	3,4,7,8
(dorsolateral superior frontal gyrus, middle frontal gyrus)		
**Temporoparietal**		
Superior parietal cortex	SPL	59,60, 69,70
(Superior parietal and paracentral lobules)		
Middle temporal gyrus	MTG	85,86
Superior temporal gyrus	STG	81,82
Inferior parietal cortex		61,62, 65,66
(Angular gyrus, Inferior parietal lobule)		
**Occipital**		
Calcarine cortex		43,44
Inferior occipital gyrus		53,54
Superior occipital gyrus		45,46
**Supplementary Regions**		
Anterior cingulate gyrus	ACC	31,32
Posterior cingulate gyrus	PCC	35,36
Insula		29,30
Heschl's gyrus		79,80
Rolandic cortex (pre- and post-central gyri)		1,2,57,58
Temporal pole		83,84,87,88
Precuneus		67,68
Supplementary motor area	SMA	19,20
Putamen		73,74
Caudate		71,72
Pallidum		75,76
Thalamus		77,78

*Left and right hemisphere regions are indicated by odd and even numbers, respectively*.

### Statistical Analyses

#### Age-Related Changes in ICC- and TSA-Values

The effect of age on ICC and TSA estimates was assessed at both the individual and group level. At the individual level, correlations of age with ICC, TSA, and ICC-TSA coupling indices were computed (zero-order and partial correlations controlling for gender, education, and HADS depression score).

Sensitivity analyses indicated that the age of 45 years represented an optimal cutoff maximizing differences between younger and older participants on ICC and TSA and minimizing within-group variance. Group-level analyses entailed one-way ANCOVAs with age as the between-subjects variable, hemisphere as a within-subject variable, and ICC, TSA, or ICC-TSA coupling indices as the dependent variable. These models were computed separately for each of 22 anatomical regions. Gender and education were included as covariates in all analyses and models were recomputed by including HADS depression score as an additional covariate. All main ANCOVAs were evaluated at Bonferroni-adjusted *p* < 0.002. In the case of a group main effect, or group by hemisphere interaction, simple main effects of group were computed.

#### Anterior-Posterior Gradient in the Effect of Age on ICC- and TSA-Values

To examine whether the impact of age on ICC- and TSA-values varies with brain section we conducted moderated regression analyses using PROCESS, a computational tool for SPSS [(71); see also, http://www.processmacro.org]. PROCESS employs Ordinary Least Squares Regression to compute regression coefficients and corresponding bootstrapped 95% Confidence Intervals (CIs) and associated *p*-values. In the context of three sets of moderated regression models we assessed interactions between age and brain section on the dependent variable (ICC- or TSA-values) and estimated the conditional effect of age on the dependent variable at each level of the categorical moderator (prefrontal, temporoparietal, and occipital, separately for each hemisphere). Specifically, we assessed differences of prefrontal (indicated by a value of 0) with occipital regions (indicated by a value of 1), prefrontal (indicated by a value of 0) with temporoparietal regions (indicated by a value of 1), and finally of temporoparietal (indicated by a value of 0) with occipital regions (indicated by a value of 1). In these analyses, values were averaged across IFG/vmPFC/dlPFC (to form the prefrontal partition), middle temporal/superior temporal/inferior parietal/superior parietal (temporoparietal partition), and calcarine/inferior/superior occipital regions (occipital partition; see Table 1). Gender and education served as covariates. Significant interactions were followed up by tests of the effect of age within each brain partition and hemisphere, evaluated at Bonferroni-adjusted *p* < 0.004.

## Results

### Changes in Intrinsic Connectivity Associated With Age

**Figure 1:**
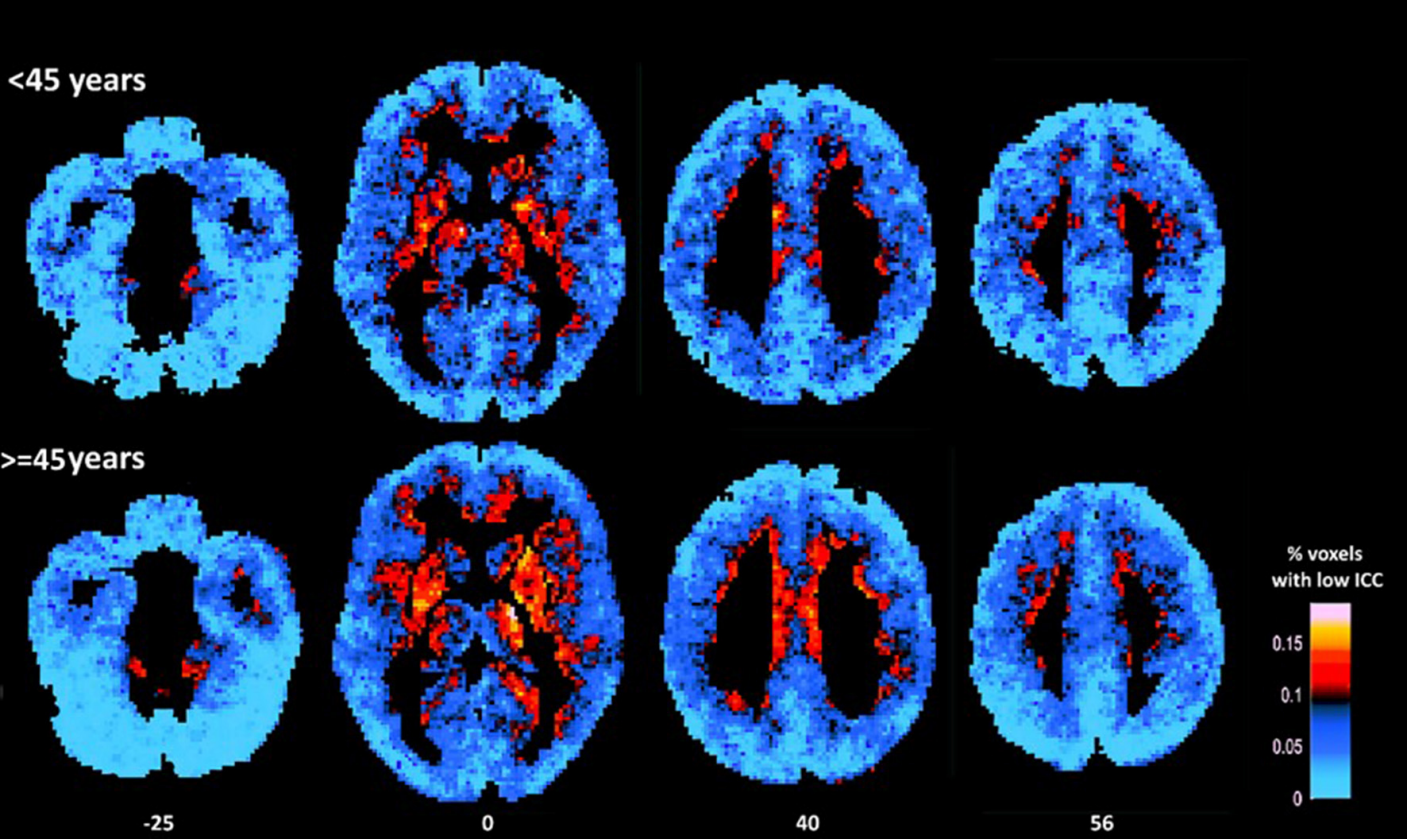
Average maps of voxels displaying relatively low ICC for younger (<45 years) and older participants (≥45 years). As a group older adults displayed higher percentage of voxels characterized by low ICC (as indicated by more hot colors) in prefrontal (medial and dorsolateral) cortices, insula, and basal ganglia (mainly the putamen) as compared to younger adults (in statistical analyses conducted at the ROI-level). Conversely, younger adults displayed lower percentage of voxels characterized by relatively low ICC in inferior occipital cortices (as indicated by lighter blue color).

Significant effects of age indicating lower ICC (e.g., higher percentage of voxels displaying relatively low ICC-values, controlling for gender and education) in older as compared to younger participants were found in IFG: *F*_(1,62)_ = 10.28, *p* = 0.002, vmPFC: *F*_(1,62)_ = 7.44, *p* = 0.01, dlPFC: *F*_(1,62)_ = 7.05, *p* = 0.01, SMA: *F*_(1,62)_ = 16.04, *p* < 0.001, PCC: *F*_(1,62)_ = 10.04, *p* = 0.002, temporal pole: *F*_(1,62)_ = 12.17, *p* = 0.001, insula: *F*_(1,62)_ = 8.49, *p* = 0.005, and putamen: *F*_(1,62)_ = 13.08, *p* = 0.001 (see Figure 1). Marginally significant trends in that direction were also found in the caudate [*F*_(1,62)_ = 6.61, *p* = 0.01; r_age−ICC_ = −0.235, *p* = 0.006] and pallidum [*F*_(1,62)_ = 7.01, *p* = 0.01; r_age−ICC_ = −0.325, *p* = 0.01]. The opposite trend (higher ICC-values among older adults) was found in the inferior occipital gyrus: *F*_(1,62)_ = 8.90, *p* = 0.004.

**Figure 2:**
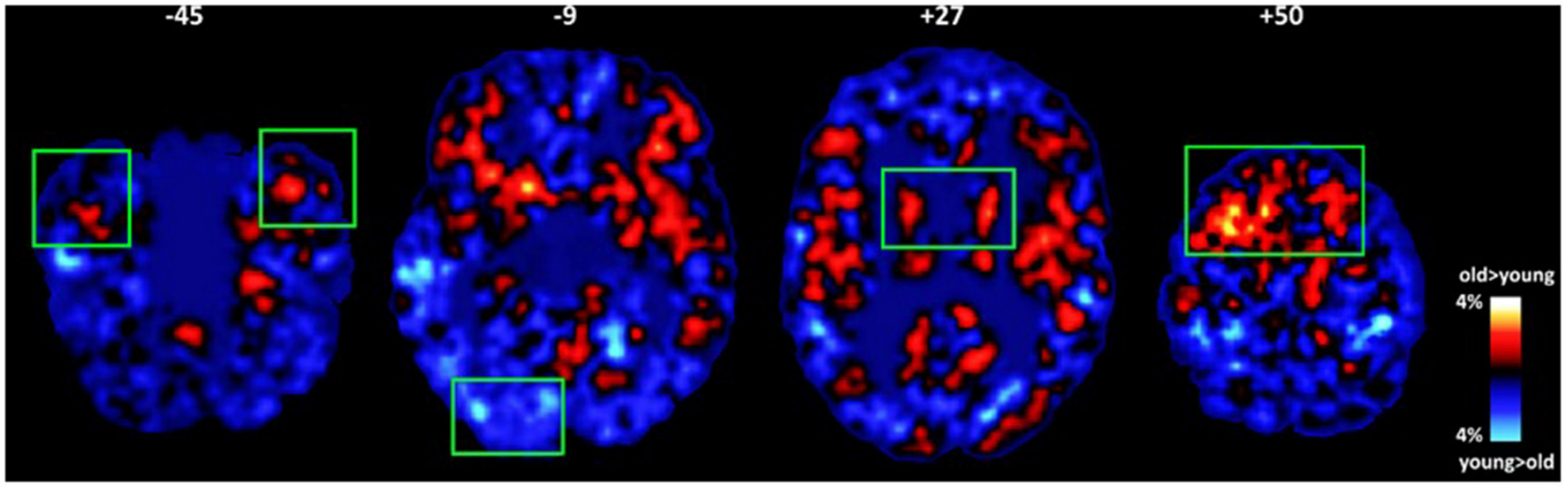
Average maps of voxels where group differences in intrinsic connectivity were found between older and younger adults (old minus young maps of voxels displaying relatively lower ICC). Significant effects of age adjusting for gender, education, and subclinical depression symptomatology are marked by green rectangles (*p* < 0.01). Older adults displayed higher percentage of voxels characterized by *low* ICC in the temporal pole, putamen, and prefrontal (medial and dorsolateral) cortices. The opposite trend was found in the inferior occipital gyrus.

In models that included gender, education, and depression symptoms as covariates, the effect of age was reduced in the majority of ROIs and remained significant (or marginally significant) in the temporal pole [*F*_(1,61)_ = 4.20, *p* = 0.05; Partialr_age−ICC_ = 0.435, *p* = 0.001], vmPFC [*F*_(1,61)_ = 6.92, *p* = 0.01; Partialr_age−ICC_ = 0.426, *p* = 0.002], putamen [*F*_(1,61)_ = 3.99, *p* = 0.05; Partialr_age−ICC_ = 0.384, *p* = 0.005], and inferior occipital gyrus [*F*_(1,61)_ = 4.08, *p* = 0.05; Partialr_age−ICC_ =-0.360, *p* = 0.009; see Figure 2].

### Changes in Perfusion Dynamics Associated With Age

**Figure 3:**
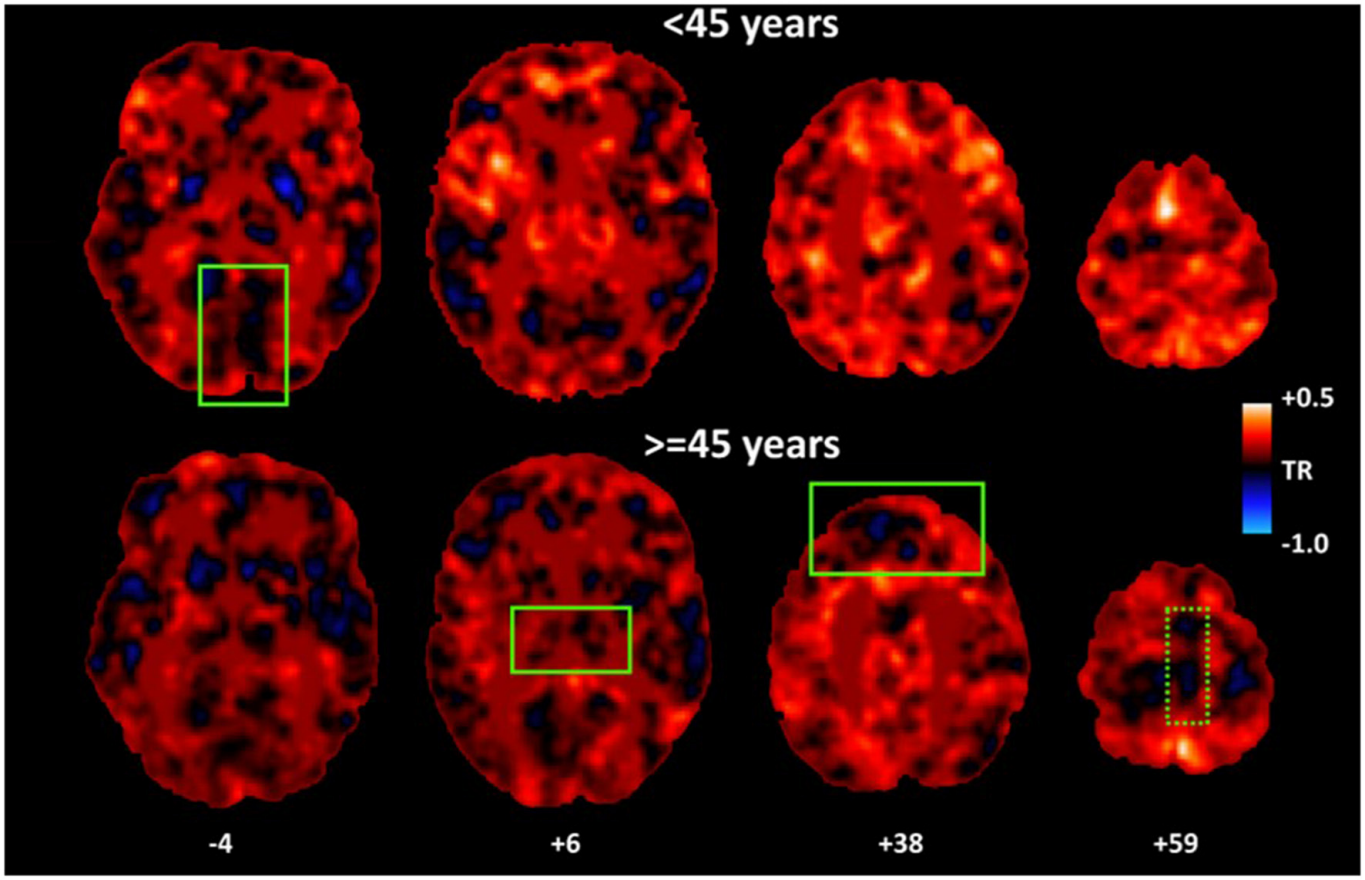
Average maps of hemodynamic lead for younger (<45 years) and older participants (≥45 years). As a group, older adults displayed higher percentage of voxels characterized by hemodynamic lead (as indicated by more cold colors) in vmPFC and thalamus as compared to younger adults. A similar effect in SMA [dotted rectangle] depended upon depression symptoms. Conversely, younger adults displayed lower percentage of voxels displaying hemodynamic lead in inferior occipital cortices.

Age-, gender-, and education-adjusted trends for higher hemodynamic lead (e.g., higher percentage of voxels displaying TSA-values <1 TR) with advancing age in SMA, vmPFC, and thalamus, accompanied by lower hemodynamic lead in the inferior occipital gyrus (see Table 2 and Figure 3). However, after adjusting for depression symptoms age-related effects were limited to the vmPFC [*F*_(1,61)_ = 6.08, *p* = 0.01; Partialr_age−TSA_ = 0.510, *p* < 0.001], and thalamus [*F*_(1,61)_ = 4.99, *p* = 0.01; Partialr_age−TSA_ = 0.52, *p* < 0.001], revealing increased hemodynamic lead with advancing age. The converse effect of aging (reduced hemodynamic lead with advancing age adjusted for depression symptoms) was found in inferior occipital cortices [*F*_(1,61)_ = 5.03, *p* = 0.01; Partialr_age−TSA_ = 0.499, *p* < 0.001]. There were no significant age effects on indices of hemodynamic lag.

**Table 2:** Age effects on intrinsic connectivity and hemodynamics.

**Anatomical region**	**Low ICC**		**Hemodynamic lead**	
	**Group effect (*p*)^a^**	**Correlation with age (*r*, *p*)^a^**	**Group effect (*p*)^a^**	**Correlation with age (*r*, *p*)^a^**
	**Old>Young**		**Old>Young**	
IFG	0.002	0.407 (0.001)		
vmPFC	0.01	0.395 (0.002)^b^	0.0012	0.535 (<0.001)^b^
dlPFC	0.01	0.387 (0.002)		
SMA	0.001	0.440 (0.001)	0.01	0.380 (0.002)
PCC	0.002	0.320 (0.01)		
Temporal pole	0.001	0.469 (<0.001)^b^		
Insula	0.005	0.398 (0.001)		
Putamen	0.001	0.508 (<0.001)		
Thalamus			0.01	0.545 (<0.001)^b^
	**Old < Young**		**Old < Young**	
Inferior occipital gyrus	0.001	−0.544 (<0.001)	0.001	−0.518 (<0.001)^b^

a *Controlling for gender and education in years*.

b *Remained significant (p <0.002) after controlling for depression symptoms*.

### Changes in the Coupling of Perfusion Dynamics With Intrinsic Connectivity Associated With Age

**Figure 4:**
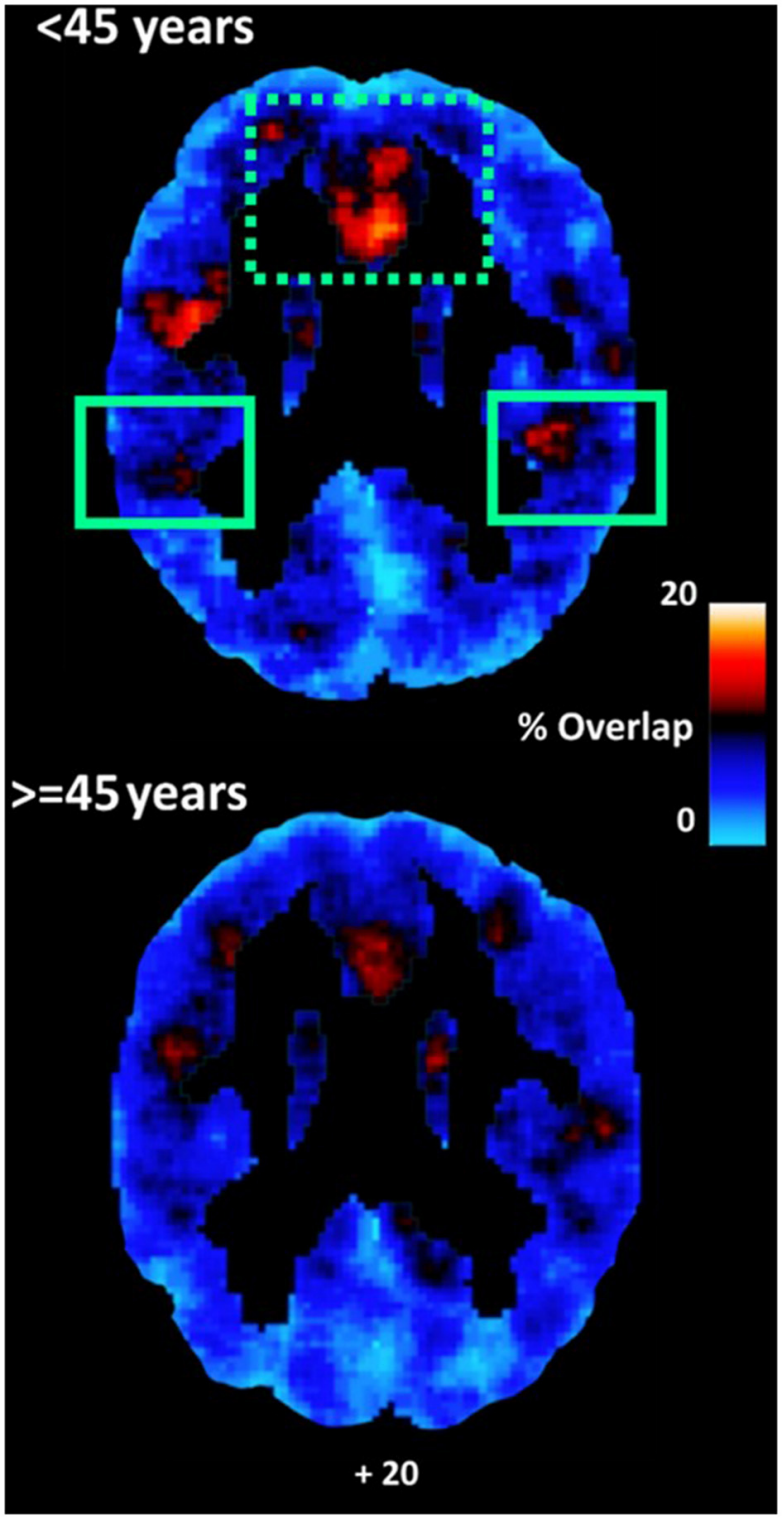
Average maps of coupling between voxels displaying relatively lower ICC-values and voxels displaying hemodynamic lag for younger (<45 years) and older participants (≥45 years). Coupling was higher among younger as compared to older adults in the inferior parietal lobule (adjusted for depression symptoms). A similar effect in vmPFC [dotted rectangle] depended upon depression symptoms.

Controlling for gender and education, significant effects of age indicating lower ICC-TSA coupling (as indicated by percent overlapping voxels displaying both relatively lower ICC-values and hemodynamic lag) in older as compared to younger participants were found in vmPFC [*F*_(1,62)_ = 10.64, *p* = 0.002; r_age−ICC/TSA_ = −0.433, *p* < 0.001] and inferior parietal cortex [*F*_(1,62)_ = 17.94, *p* < 0.001; r_age−ICC/TSA_ = −0.390, *p* = 0.002; see Figure 4]. There effects were paralleled by trends found within maps of high ICC-hemodynamic lead coupling which, however, did not reach significance.

In models that included gender, education, and depression symptoms as covariates, the effect of age was reduced and remained marginally significant in the inferior parietal cortex [*F*_(1,61)_ = 18.29, *p* < 0.001; Partialr_age−ICC/TSA_ = −0.362, *p* = 0.009].

### Anterior-Posterior Gradient in the Effect of Age on Intrinsic Connectivity

As shown by the results of moderated regression analyses presented in Table 3, significant age by region (anterior vs. posterior) interactions were found on several occasions. Inspection of unstandardized regression coefficients indicates that age-related reductions in ICC were greater in prefrontal (indicated by positive regression coefficients on percentage of voxels displaying relatively lower ICC) as compared to the occipital lobes bilaterally (indicated by negative regression coefficients). A similar effect was noted for prefrontal vs. temporoparietal ICC in the left hemisphere. These effects persisted after controlling for gender and depression. Region did not moderate the effect of age on hemodynamic lead- or lag-values (*p* > 0.5). It should be noted that correlations between age and ICC in primary somatosensory, auditory, and motor areas were negligible (|*r*| < 0.15).

**Table 3:** Unstandardized regression coefficients (*b*-values) reflecting the contribution of age to ICC-values in brain sections displaying significant anterior-posterior gradient.

**Brain partition**	**Hemisphere**	**b**	**SE**	**p**
*Prefrontal vs. Occipital*	*Left*	–*0.0023*	*0.0005*	*<0.001*
Prefrontal		0.0014	0.0003	**<0.001**
Occipital		−0.0009	0.0004	0.01
*Prefrontal vs. Occipital*	*Right*	–*0.002*	*0.0005*	*<0.001*
Prefrontal		0.0014	0.0004	**<0.001**
Occipital		−0.0006	0.0003	0.08
*Prefrontal vs. Temporoparietal*	*Left*	–*0.0013*	*0.0005*	*0.007*
Prefrontal		0.0013	0.0003	**<0.001**
Temporoparietal		0.00001	0.0004	0.9

*Gender and education served as covariates. Significant conditional effects of age (at Bonferroni-adjusted p < 0.004) are shown in bold*.

### Hemisphere Differences in Global Connectivity and Hemodynamic Status

As indicated by main effects of hemisphere (in the absence of hemisphere by age interactions), higher ICC-values were found in several left hemisphere ROIs as compared to homologous right hemisphere regions, namely SMA: *F*_(1,62)_ = 11.02, *p* = 0.002, ACC: *F*_(1,62)_ = 12.25, *p* = 0.001, calcarine cortex: *F*_(1,62)_ = 43.93, *p* < 0.001, temporal pole: *F*_(1,62)_ = 19.17, *p* < 0.001, and precuneus: *F*_(1,62)_ = 16.38, *p* < 0.001.

Hemodynamic status was in partial agreement with intrinsic connectivity: there was a trend for higher hemodynamic lead in the right as compared to the left hemisphere in SMA: *F*_(1,62)_ = 4.29, *p* = 0.04, and inferior occipital gyrus: *F*_(1,62)_ = 5.14, *p* = 0.02.

## Discussion

The results of the study can be summarized as follows: (a) Several regions predominantly within the lateral and medial frontal cortices, PCC, basal ganglia, insula, and temporal pole displayed age-related significant reductions in intrinsic connectivity. SMA, superior frontal cortices, and thalamus were also characterized by age-related increased hemodynamic lead. The opposite effects (increased intrinsic connectivity and reduced hemodynamic lead with aging) was observed in the occipital lobe. (b) Age-related reductions in intrinsic connectivity were greater in anterior (prefrontal) as compared to posterior (occipital and temporoparietal) cortices, in line with predictions derived from the retrogenesis hypothesis. (c) Although these effects were not affected by gender or participant formal education, they depended considerably upon self-reported depression symptoms which also increased with age (see, also, Supplementary Material).

In comparing the present findings with previous work it is important to note that functional connectivity was estimated using a whole-brain voxel-based approach to overcome the problem of setting a priori seeds and thresholds for connectivity indices (e.g., correlations between time series). Few, very recent studies have employed a similar approach to ours. Specifically, these results are in partial agreement with a recent report of reduced ICC in prefrontal regions, insula, and temporal pole in older adults paralleled by increases in inferior occipitotemporal and occipitoparietal regions (49). Reduced ICC in the insula, dlPFC (middle and inferior frontal gyri), SMA, cingulate gyrus, and putamen has also been found among healthy older adults by Bagarinao et al. (50). It should be noted, though, that in earlier studies concurrent depression symptoms were not taken into account in assessing age-related effects thus potentially affecting the degree of agreement with the present results.

The majority of studies on healthy aging has employed a network-based approach, rendering comparisons with the present work problematic. There are however some notable similarities. Thus, vmPFC is considered a key component of the DMN (72) whereas the insula, ACC, and adjacent frontal opercular region are components of the ventral attention network (73). Decreased connectivity within both networks with advancing age has been reported using various methodologies (42, 44–47).

In the present study significant changes, mainly, in intrinsic connectivity were noted after approximately the age of 45 years. Similar trends have been found in samples comparable in age range as ours using MTR (74) and transverse relaxation rate (25). The estimated age at which maximum Myelin Water Fraction values were reached is in agreement with Bartzokis et al. (25) and Westlye et al. (75), who reported peak transverse relaxation rates in prefrontal white matter at 32 years and peak FA-values in major cortico-cortical tracts traversing the temporal lobe at 28–29 years, respectively. In our previous study utilizing T2 relaxometry the earliest significant reduction in myelin content in the frontal lobe was noted after the 5th decade while there is evidence that deep lobar myelin content continues to increase through the third decade of life (37). Interestingly, myelin content reduction was evidenced in the occipital NAWM as well (albeit less steeply), while intrinsic connectivity of corresponding cortices appears to change very little according to the present data.

The present findings complement our previous report demonstrating steeper age-related reduction in myelin content (indexed by Myelin Water Fraction) within the prefrontal NAWM, followed by lateral-posterior temporal, parietal, and occipital lobes (37). These results were obtained using the T2 relaxometry which appears to be more sensitive to myelin content than other techniques such as MTR and DTI. The current findings further suggest that changes in myelin content are accompanied by comparable rates of reduction in intrinsic connectivity, displayed by cortex served by underlying white matter, and, also, higher hemodynamic lead, both predominating at prefrontal as compared to occipital regions. Taken together, relaxometry and resting-state fMRI findings are consistent with implications derived from the retrogenesis hypothesis, according to which white matter regions that complete their myelination early in human development (such as the occipital cortices) are less susceptible than later-myelinating regions to age-related demyelination in late adulthood (35, 36) and this is possibly related to compensatory hemodynamic mechanisms.

Although the rate of decline in myelin content did not vary between hemispheres, in agreement with previous studies that explicitly tested this hypothesis using DTI (76) and T2 relaxometry (37), there was evidence of higher global functional connectivity of several left hemisphere regions as compared to homologous regions in the right hemisphere (SMA, cingulate gyrus, cuneus, precuneus, visual cortex, amygdala and parahippocampal gyrus, and temporal pole). This finding is in agreement with the few small-scale studies that explicitly examined hemisphere differences in deep lobar white matter characteristics using FA (38) and MTR (39).

To our knowledge this is the first study investigating age-related hemodynamic differences, using TSA analysis of resting state fMRI data. This new method has been used previously to assess hemodynamics in CNS diseases, mainly involving hemodynamic impairment in chronic hypoperfusion and acute/subacute stroke (51–53, 56, 77). In these studies, hemodynamic lag times, representing BOLD signal delay, are thought to reflect background vascular delay and indicated hypoperfused tissue. ASL studies revealed hypoperfusion with advancing age in healthy persons, by demonstrating global or regional gray matter age-related CBF reductions predominantly in frontal lobes, although increased CBF-values have, also, been reported in different areas (17, 21). On the contrary, there were no age-related hemodynamic lag times, indicating severe hypoperfusion, in the current study. This could be partially explained by: (a) the different aspects of hypoperfusion reflected in the delay in BOLD fluctuations in rs-fMRI and in the CBF reduction estimated by ASL, (b) the relative low temporal resolution of rs-fMRI that interferes with the detection of minor perfusion decreases, (c) the different principles of hemodynamics between the two techniques: CBF changes in ASL are measured through the transit delays of the spin labels through arteries and arterioles (78), whereas BOLD contrast in TSA is weighted toward the venous drainage system (54, 60).

Several studies have shown a close relationship between BOLD delay and perfusion indices, other than CBF, that have been measured by dynamic susceptibility-contrast perfusion-weighted imaging (DSC-PWI). These are: Tmax (51, 79, 80), MTT (53, 79), and time to peak (TTP; 56, 77). Ni et al. (77), using the TSA approach, detected hypoperfusion areas in patients with intracranial large-vessel occlusion or severe stenosis without collaterals, which were comparable to the TTP delay areas detected by DSC-PWI. Conversely, patients without obvious vascular lesions in MR Angiography showed neither TTP nor TSA time delay.

Direct comparisons of the diagnostic performances of BOLD delay and ASL for detecting hypoperfusion are very rare. Siegel et al. (57) in a longitudinal study of hemodynamic lag in 130 patients with stroke and 30 healthy subjects found that areas with lag >2 s showed significantly lower CBF-values, while CBF in areas of hemodynamic lag <1 s was, on average, 92.46% of that of controls. Even more, areas in which lag recovers do not necessarily return to normal perfusion. Staffaroni et al. (81) in an ASL study demonstrated an annual global gray matter CBF decline of just of 0.66 ml/100 g/min in healthy subjects, with lack of association between age and CBF changes. It is worth noting that the temporal resolution of rs-fMRI is relatively low (2.3 s in the current study), possibly responsible for insufficient depiction of minor perfusion decreases. Acquiring BOLD-fMRI at a higher temporal resolution, may improve the assessment of low grade hypoperfusion.

Our findings of age-related increased hemodynamic lead in SMA and reduced hemodynamic lead in the inferior occipital gyrus, are in partial agreement with the faster cerebrovascular responses in the primary motor and somatosensory cortex, compared to temporoparietal and inferior occipital regions, demonstrated by Qian et al. (60) in elderly (>60 years) healthy participants, using TSA. In this study time shift values were different in distinct vascular territories, with the middle cerebral artery territory having the earliest blood arrival time. Several ASL and rs-fMRI studies in healthy subjects demonstrated substantial systematic variability across cerebral perfusion territories, as well as, in vascular reactivity to respiratory changes, in different cortical areas (82–84). The age-related regional hemodynamic lead in SMA found in our study could, also, be partially explained by the representation of the respiratory muscles in motor cortex (85) and the increased respiratory rate by aging (86). Even more, the variability in vascular reactivity may enhances with aging, due to increasing prevalence of cardiovascular co-morbidity.

In the current study there was also evidence of reduced *coupling* between perfusion dynamics and intrinsic connectivity with age, in prefrontal (especially in the left hemisphere) and parietal regions (especially in the right hemisphere). These findings are in partial agreement with a recent study by Galiano et al. (49) who employed ASL to measure age-related CBF and ICC changes. In young healthy persons connectivity strength, within all functional networks, significantly correlated with regional increases in CBF-BOLD coupling strength (62). With aging, cerebrovascular damage and hypoperfusion may be accompanied by a compensatory increase in regional oxygen extraction (87) that prevents neural activity compromise and resulting in reduced neurovascular coupling (88).

In interpreting the present findings and comparing them with previous studies, it is important to note that older adults were deemed as cognitively intact via extensive neuropsychological assessment. Despite considerable variability in performance on individual cognitive tasks, none of the participants met the IWG criteria for Mild Cognitive Impairment (67). However, we did not exclude participants who reported subjective cognitive complaints. The latter are quite common even in non-clinical samples and may affect measures of brain structure and function even when objective cognitive impairment cannot be detected through formal neuropsychological testing as in the present study (89, 90).

## Limitations

The aforementioned findings should be interpreted with caution, however, in view of certain limitations. Importantly, the present results rely on cross-sectional data and are subject to sampling bias and cohort effects. Moreover, perfusion indices were derived from the analysis of time-lags (or leads) in resting-state recordings across brain voxels. These measures of time shift in the phase of low-frequency BOLD oscillations are presumed to reflect hemodynamic transfer lag or lead times and, at least, in part serve as indirect indices of regional brain perfusion (54). Indices of hemodynamic lag have been validated in cases of severe brain ischemia and have been shown to represent hypoperfused tissue (52, 56, 57), although the significance of hemodynamic lead has not been explored systematically. Finally, we did not assess potential dependencies between time-lag indices and functional connectivity measures.

There are, also, some additional limitations in the current study. Although patients with cognitive decline or neurological diseases were excluded, age-related asymptomatic cardiovascular comorbidities and respiratory changes may affect the estimations of age effects in functional connectivity and compromise the validity of TSA technique for the detection of hemodynamic changes. Long scanning time of rs-fMRI makes it vulnerable to head motion. Nevertheless, its lower cost, high stability and lack of necessity for paramagnetic contrast media make the technique attractive for the non-invasive estimation of brain hemodynamics. Scanning time reduction and motion correction techniques could manage head motion effectively and further facilitate the application of rs-fMRI for the estimation of hemodynamic changes in clinical practice.

## Conclusion

In conclusion, age-related changes in both hemodynamic status and functional connectivity were estimated in the current study, by using rs-fMRI and the TSA technique. Several regions—in their majority parts of the DMN and salience networks—displayed age-related intrinsic connectivity reductions, and increased hemodynamic lead was demonstrated in prefrontal, medial premotor cortices, and thalamus, while impaired neurovascular coupling was found in prefrontal and parietal regions. In parallel, age-related reductions in intrinsic connectivity were greater in anterior as compared to posterior cortices, supporting the retrogenesis hypothesis. These effects were in part explained by increasing self-reported depression symptoms with age.

## Data Availability Statement

The raw data supporting the conclusions of this article will be made available by the authors, without undue reservation.

## Ethics Statement

The studies involving human participants were reviewed and approved by Clinical Research Ethics Board of the University Hospital of Heraklion. The patients/participants provided their written informed consent to participate in this study.

## Author Contributions

EK and NS processed and analyzed the imaging data and wrote the manuscript. TM conducted the MRI procedure and acquired the imaging data. IZ and SP recruited and assessed the participants and reviewed versions of the manuscript. EP designed the study, wrote, and reviewed the manuscript. All authors contributed to the article and approved the submitted version.

## Conflict of Interest

The authors declare that the research was conducted in the absence of any commercial or financial relationships that could be construed as a potential conflict of interest.
