# Supplementary material for: Evidence of Age-Related Hemodynamic and Functional Connectivity Impairment: A Resting State fMRI Study
Source: Front Neurol. 2021 Mar 23;12:633500. doi: 10.3389/fneur.2021.633500 (PMC8021915; doi:10.3389/fneur.2021.633500)
Supplement: Supplementary file 1 [file Data_Sheet_1.docx]

**EVIDENCE OF AGE-RELATED HEMODYNAMIC AND FUNCTIONAL CONNECTIVITY IMPAIRMENT: A RESTING STATE fMRI STUDY**

**Supplementary Material**

A potential confounder in previous studies of age-related changes in perfusion and functional connectivity is depression symptomatology which, even if undiagnosed (subclinical) may account for disturbances in functional connectivity. Whereas seed-based analyses have identified reductions in connectivity mainly in prefrontal and inferior parietal regions (1,2), whole-brain approaches have reported positive correlations between degree of connectivity in inferior parietal, rostral and ventral prefrontal cortices with higher levels of self-reported depression symptoms (3). Accordingly, in supplementary analyses we examined the role of subclinical depression symptoms as a confounding factor contributing to apparent changes in brain function with age. Specifically, we assessed correlations between HADS depression scores and ICC/TSA values in selected ROIs (vmPFC, dlPFC, ACC, PCC, insula, amygdala, hippocampus separately in each hemisphere; evaluated at p<.0035). The older group comprised a higher percentage of persons scoring > 7 points on the HADS depression subscale (n= 16 [38.0%] vs. n=4 [18.2%], p=.1), although none of the participants reported having visited a mental health professional or receiving psychotropic medications. Moreover, older adults on average scored higher on the HADS Depression subscale than younger adults: 3.58, SD=1.83 vs. 6.61, SD=2.16, p<.001.

Analyses revealed significant (positive) correlations between HADS Depression score and percentage of voxels displaying hemodynamic lead in the left vmPFC (r=.44, p=.002), and left ACC (r=.42, p=.002). Self-reported symptoms of depression also correlated positively with percentage of voxels displaying relatively lower ICC in the left PCC (r=.48, p<.001). This finding is in agreement with Liao et al. (2017) (4) who estimated whole-brain CBF in a group of older adults with late onset depression. We also found reduced intrinsic connectivity in the PCC to be associated with more severe (subclinical) depression symptoms independent of age and gender. Unfortunately, direct comparisons with the few previous studies that explored functional brain connectivity in relation to subclinical depression symptoms are precluded by methodological differences given that only one study adopted a whole-brain voxel-wise connectivity approach like ours (3). However, they studied young adults and relied on a graph metric (Degree) to estimate functional connectivity. Differences in the neural substrates of primary (early-onset) and late-onset subclinical depression symptoms notwithstanding, reduced connectivity of the PCC is often reported in the former condition (5,6).

**References**

1. Hwang JW, Egorova N, Yang XQ, Zhang WY, Chen J, Yang XY, Hu LJ, Sun S, Tu Y, Kong J. Subthreshold depression is associated with impaired resting-state functional connectivity of the cognitive control network. Transl Psychiatry. 2015 Nov 17;5(11):e683.
2. Schultz, D. H., Ito, T., Solomyak, L. I., Chen, R. H., Mill, R. D., Anticevic, A., & Cole, M. W. (2018). Global connectivity of the fronto-parietal cognitive control network is related to depression symptoms in the general population. Network Neuroscience (Cambridge, Mass.), 3(1), 107–123.
3. Gao, C., Wenhua, L., Liu, Y., Ruan, X., Chen, X., Liu, L., Yu, S., Chan, R. C., Wei, X., & Jiang, X. (2016). Decreased Subcortical and Increased Cortical Degree Centrality in a Nonclinical College Student Sample with Subclinical Depressive Symptoms: A Resting-State fMRI Study. Frontiers in Human Neuroscience, 10, 617.
4. Liao, W., Wang, Z., Zhang, X., Shu, H., Wang, Z., Liu, D., & Zhang, Z. (2017). Cerebral blood flow changes in remitted early- and late-onset depression patients. Oncotarget, 8(44), 76214–76222.
5. Dong D, Li C, Ming Q, Zhong X, Zhang X, Sun X, Jiang Y, Gao Y, Wang X, Yao S. Topologically state-independent and dependent functional connectivity patterns in current and remitted depression. J Affect Disord. 2019 250:178-185.
6. Zhu Y, Wang D, Liu Z, Li Y. Aberrant topographical organization in default-mode network in first-episode remitted geriatric depression: a graph-theoretical analysis. Int Psychogeriatr. 2018 30(5):619-628.
